# Direct Energy Deposition of TiAl for Hybrid Manufacturing and Repair of Turbine Blades

**DOI:** 10.3390/ma13194392

**Published:** 2020-10-01

**Authors:** Silja-Katharina Rittinghaus, Janett Schmelzer, Marcus Willi Rackel, Susanne Hemes, Andreas Vogelpoth, Ulrike Hecht, Andreas Weisheit

**Affiliations:** 1 Fraunhofer Institute for Laser Technology (FhG-ILT), 52074 Aachen, Germany; andreas.vogelpoth@ilt.fraunhofer.de (A.V.); andreas.weisheit@ilt.fraunhofer.de (A.W.); 2 Otto-von-Guericke-Universität Magdeburg, Institut für Werkstoff und Fuegetechnik, 39106 Magdeburg, Germany; janett.schmelzer@ovgu.de; 3 Helmholtz-Zentrum Geesthacht, 21502 Geesthacht, Germany; marcus.rackel@hzg.de; 4 Access e.V, 52072 Aachen, Germany; s.hemes@access-technology.de (S.H.); u.hecht@access-technology.de (U.H.)

**Keywords:** titanium aluminides, additive manufacturing, microstructure, phase distribution, mechanical properties

## Abstract

While repair is mainly used to restore the original part geometry and properties, hybrid manufacturing aims to exploit the benefits of each respective manufacturing process regarding either processing itself or resulting part characteristics. Especially with the current implementation of additive manufacturing in the production of TiAl, turbine blades for both hybrid manufacturing and repair new opportunities are enabled. One main issue is the compatibility of the two or more material types involved, which either differ regarding composition or microstructure or both. In this study, a TNM^TM^-alloy (Ti-Nb-Mo) was manufactured by different processes (casting, forging, laser additive manufacturing) and identically heat-treated at 1290 °C. Chemical compositions, especially aluminum and oxygen contents, were measured, and the resulting microstructures were analyzed with Scanning Electron Microscopy (SEM) and High-energy X-ray diffraction (HEXRD). The properties were determined by hardness measurements and high-temperature compression tests. The comparison led to an overall assessment of the theoretical compatibility. Experiments to combine several processes were performed to evaluate the practical feasibility. Despite obvious differences in the final phase distribution caused by deviations in the chemical composition, the measured properties of the samples did not differ significantly. The feasibility of combining direct energy deposition (DED) with either casting or laser powder bed fusion (LPBF) was demonstrated by the successful build of the dense, crack-free hybrid material.

## 1. Introduction

Due to suitable high-temperature properties, such as high specific high-temperature strength (100–150 MPa cm^3^/g), high specific rigidity (35–42 GPa cm^3^/g), high oxidation, and creep resistance, intermetallic γ titanium aluminide alloys with a density of ρ = 3.9–4.1 g/cm^3^ are considered to be substitutes for nickel-based superalloys with approximately twice the density (ρ = 7.9–8.5 g/cm^3^) [1,2]. The limited ductility and fracture toughness of the material [3,4], as well as the high reactivity of titanium, especially with oxygen, water, and nitrogen, are challenging both for the use and the production of titanium aluminide components. Applications are mainly in the aerospace industry and, in some cases, in the automotive and energy sectors [1,3]. One of the main applications is the use of low-pressure turbine blades at an operating temperature of approx. 700 °C. The best-known alloys currently in service are GE4822 (Ti-48Al-2Nb-2Cr, at.%), a two-pha TiAl alloy used by GE Aviation/Avio Aero in the GE90, CFM International LEAP (formerly LEAP X) and GEnX engines, and the TNM-B1 (B1 = 0.1 wt.-% B) [5]. TNM^TM^ is a group of forging alloys fully solidified through the β phase and protected by MTU Aero Engines AG. Leistritz Turbinentechnik GmbH uses it to manufacture low-pressure turbine (LPT) blades [5], which are installed in the geared turbofan PW1110G. The target element distribution of the main alloy elements is adjusted according to the desired distribution of the three phases—α2, γ, and β/β0(B2) [1]—and is in the range Ti(42-44)Al-(3-5)Nb-(0.1-2)Mo-(0.1-0.2)B (in at.%).

The alloy investigated in this paper is designated TNM-B1. The nominal composition may differ, and the one used here is Ti-43.5Al-4Nb-1Mo-0.1B (in at.%) [6]. The density is 4.16 g/cm³. The alloy solidifies via the β phase by means of β phase stabilizing elements, such as Mo and Nb [7]. Diffusion processes in the α2 and γ phase are crucial for heat treatment results and slowed down by Nb. An increase in the activation energy of diffusion in these phases is achieved by the addition of Mo [8]. Both serve to improve the creep properties. Boron is added in the range of 0.1–1 at% due to its grain refining and texture-reducing effect [2,4,7]. The addition of boron leads to the formation of stable Ti-boride precipitates, which also inhibit grain growth during heat treatment.

Casting and forging processes are established for production, which are very complex due to the low ductility and fracture toughness as well as the high oxygen affinity of the intermetallic material. The industry’s interest in the tool-free additive production of titanium aluminide components is, therefore, great. Functional tests, e.g., at GE Avio Aero with blades manufactured by additive electron beam melting (EBM), are already being carried out [9]. Other approaches in research include powder metallurgy (PM) via hot isostatic pressing (HIP) [10], metal injection molding (MIM) [11], or spark plasma sintering (SPS) [12]. With the increasing use of γ-TiAl in aircraft turbines, the need for repair or modification solutions will increase, irrespective of the original production route of the components concerned. Currently, the research is performed on transient liquid phase (TLP) bonding for the repair of γ-TiAl, using Fe or Ni as melting point depressing elements (MPD) [13]. Furthermore, in the recently completed LuFo-project (FKZ 20T1526A), the diffusion welding of TiAl was investigated with, again, the aim of the repair.

With direct energy deposition (DED), components can not only be built up additively but also be repaired near net-shape. The suitability of DED as an additive manufacturing process for coating and repair tasks has already been proven for numerous material groups (nickel-based alloys, steel materials, etc.). Especially the ability to build on 3D surfaces enables DED as a potential method to individualize parts manufactured via a different route.

Crack-free processing of titanium aluminides with DED is possible by preheating the substrate during the process [14,15,16,17,18]. The temperature of the substrate or the already built-up volume required to produce crack-free traces must be in the brittle-ductile transition range (550–800 °C) for Ti-based alloys [19]. The actual preheating temperature required depends on the alloy, the geometry, and the process parameters. To protect the melt from oxidation, the process zone is shielded locally with inert gas [15] or a global inert gas atmosphere [17,20,21,22,23,24] with < 100 ppm [20] or even smaller amounts of O [25]. The oxygen content after DED has been measured in [26,27] at approx. 500–1200 ppm with complete shielding. The influence of process conditions and process parameters on the oxygen content in the material produced has hardly been investigated so far [27], and the contents of <1000 ppm O in DED GE4822 and TNM-B1 have been documented in some publications [15,25,28].

According to [29], phase transformations can be achieved for TNM at temperatures between 1230 °C and 1300 °C, depending on the composition and thus, for example, the aluminum content. In [30], results are published on the influence of heat treatment temperature, duration, and cooling rate. A typical composition for a TNM type alloy (MTU/Pratt Whitney) is approximately 79% γ, 25% α2, and 5% β [31]. However, the heat treatment methods applied in the literature vary widely. Thus, a direct comparison of the microstructure and properties is hardly possible but crucial to determine whether materials from the same alloy are compatible, as the final part can be treated only as a whole.

Following this approach, samples in this work produced with different manufacturing methods, including conventional as well as additive manufacturing, were identically heat treated. The material was examined regarding microstructure and properties, taking into account the respective chemical composition. It was experimentally demonstrated that DED combined with cast and laser powder bed fusion (LPBF) resulted in dense, crack-free samples, which were analyzed as well. The properties of γ-TiAl produced with DED in the investigations carried out corresponded overall to those achieved with processes used to produce components made of γ-TiAl. DED was, therefore, suitable as a process for processing γ-TiAl. However, the oxygen content was still above the critical value of 1000 ppm, which reduced the ductility, especially at room temperature, and thus made reworking and assembly of components even more difficult.

## 2. Materials and Methods 

### 2.1. Materials

The powder materials used in this work, made of the alloy TNM-B1, were produced by TLS Technik GmbH Spezialpulver KG (Bitterfeld-Wolfen, Germany) via the EIGA process with argon gas atomization. The used particle size distribution was 20–90 µm (DED) and 20–80 µm (LPBF), respectively. Since Al and O have a significant influence on the microstructure evolution, the content of both elements was measured for the two powder batches. The powder contained 820 ppm O and 29.5 wt.-% Al (DED) and 850 ppm O and 29.3 wt.-% Al (LPBF). DED samples were built on similar TNM substrate material with a thickness of 4 mm (produced by Access e.V., Aachen, Germany). The substrates were produced by investment casting and were used in the non-heat-treated cast state. LPBF samples were built on Ti6Al4V material.

### 2.2. Additive Manufacturing

#### 2.2.1. Direct Energy Deposition (DED) of TiAl

Tests for DED were carried out on a 3-axis CNC portal system in a laboratory setup. A 2 kW diode laser of the type LDF2000 (λ = 1025 and 1064 nm) from Laserline GmbH (Muelheim-Kaerlich, Germany) was used as a laser beam source. As for focusing optic, an optical system of the OTS 5 series from Laserline GmbH (focal length 182 mm) was used. The powder was conveyed by a conveyor from GTV Verschleißschutz GmbH (Luckenbach, Germany). The process parameters used for DED are listed in Table 1.

The complete experimental setup was located inside a protective gas chamber (glovebox), which was closed completely airtight and filled with Argon 4.6 (50 ppm O). Preheating up to 900 °C was conducted by induction heating.

#### 2.2.2. Laser Powder Bed Fusion (LPBF) of TiAl

The system used was a laboratory LPBF system equipped with a multi-mode fiber laser with a maximum laser power of 500 W. The focus diameter of the beam was 100 µm. Argon was used as protective gas. The oxygen concentration in the process chamber was < 10 ppm. The process parameters are listed in Table 2.

The system was equipped with an induction preheating system, with which the substrate could be heated up to 1600 °C. The preheating temperature applied was 900 °C.

### 2.3. Heat Treatment

Heat treatment was performed at 1290 °C for one hour, followed by furnace cooling. The cooling rate was approximately 4 K/min. To minimize surface oxidation, a continuous argon gas stream of 300 L/h was used.

### 2.4. Analytical Methods

#### 2.4.1. Chemical Composition

Oxygen contents were determined with carrier gas hot extraction. Aluminum and other elements were measured with ICP-OES, according to DIN 51008-2 and DIN 51009.

#### 2.4.2. Microstructure and Phase Analysis

Samples for microstructure analysis were cut from the build-up volumes, and the cross-sections were prepared parallel to the build direction (z). A ZeissULTRA55 SEM (Carl Zeiss Microscopy GmbH, Jena, Germany) with field-emission gun (= FE-SEM) was used for imaging. An Oxford-TETRA-BSE (= back-scattered-electron) detector (Oxford Instruments, Abingdon, UK) was used for micrographs, and the measurements were made at 15 kV.

High-energy X-ray diffraction (HEXRD) measurements were performed at DESY (Deutsches Elektronen-Synchrotron), Hamburg, Germany. The High-Energy Materials Science Beamline (HEMS/P07) operated by Helmholtz-Zentrum Geesthacht at the PETRA III synchrotron radiation facility was used. The specimens were measured in transmission with a beam size of 1 mm by 1 mm, photon energy of 100 keV (λ = 0.1240 Å), and an exposure time of 0.1 s. The Debye-Scherrer diffraction rings were recorded on a Perkin Elmer XRD 1622 flat panel detector (PerkinElemer Inc., Santa Clara, CA, USA). For calibration, a measurement with a standardized lanthanum hexaboride (LaB_6_) powder was used in order to calculate the instrumental parameters, the beam center, and the sample-detector distance. The phase fractions were calculated with the Rietveld analysis software package MAUD (Version 2.92, L. Lutterotti, University of Trento, Italy). 

#### 2.4.3. Mechanical Properties

Vickers hardness was tested with a load of 30 kgf and a loading time of 15 s. Test specimens for compression tests of the dimensions 5 × 3 × 3 mm³ were cut out of larger samples by eroding. The tests were carried out parallel to the build direction. Compression tests were performed uniaxially on a Zwick Roell Z100 (ZwickRoell GmbH & Co. KG, Ulm, Germany), equipped with a furnace from Maytec Mess- und Regeltechnik GmbH (Singen, Germany) at 700 °C under argon. The speed during compression tests was 10^−4^ mm/s until breakage.

## 3. Results

### 3.1. Chemical Composition

In Table 3, the chemical compositions of the samples used for tests are listed. Cast and forged samples were intentionally selected to have a higher oxygen content than technically possible so that the oxygen contents were similarly high for all samples, and the influence of the manufacturing route on the phase formation after heat treatment could be investigated. Significant differences can be seen in the aluminum content, which was at around 0.4 wt.% lower for LPBF, cast, and forged material than in the DED sample.

In comparison with LPBF, the Al content of the powder material used for DED was already higher (29.5 and 29.3 wt.-%, respectively). Additionally, the aluminum loss due to evaporation was likely lower due to the lower mean melt temperature. For forged material, no further influence of the manufacturing process on the composition was given as no molten phase was involved in the process.

### 3.2. Microstructure and Phases

#### 3.2.1. Microstructure

**Figure 1:**
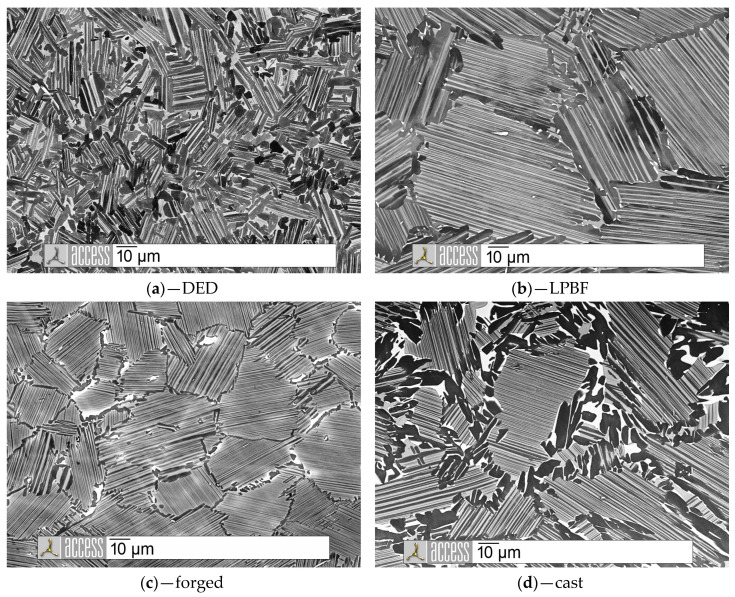
SEM images of heat-treated TNM (**a**) manufactured by DED; (**b**) manufactured by LPBF [32]; (**c**) manufactured by forging; (**d**) manufactured by casting.

In Figure 1, SEM images of the investigated material samples in the heat-treated condition are shown. All samples showed a density > 99.8%, determined by metallographic analysis, and were free of cracks. The microstructure consisted of lamellar grains (α_2_+γ colonies), inter-crystalline γ grains, and BCC-B2 (β_0_) phase fractions. The phases could be distinguished from each other by their gray-scale value: α2 (gray), γ (dark gray), and β_0_ (light gray/white).

Two transition temperatures were important in the heat treatment of γ-TiAl. The α-transus temperature T_α-transus_ (partly identical with the γ solvus temperature (T_γ-solvus_) [33]) was the temperature at which the formation of the γ phase began during cooling or at which (ordered) α and γ were dissolved during heating. Thus, by heat treatment above T_α-transus_, completely lamellar structures could be achieved on cooling. In addition, complete γ solution favored grain growth of the α phase, which was inhibited by the still existing γ phase. During heat treatment in the α_2_+γ phase region, characterized by temperatures between T_α-transus_ and the eutectoid temperature T_e_, almost lamellar to duplex structures were produced. The closer the heat treatment temperature was to T_e_, the greater the proportion of γ [34,35,36]. 

The eutectoid transformation for TNM with the nominal composition of Ti-43.5Al-4Nb-1Mo-0.1B (at.-%) occurred at T_e_ = 1160 °C, and the α to γ transformation temperature T_α-transus_ was 1250 °C. The respective transformation temperatures were sensitive to the aluminum content. Lower aluminum contents shifted the phase equilibrium towards a higher T_α-transus_. With regard to the effect of oxygen on the solid-state transformation, the ordered α_2_ phase was stabilized against the α phase at least in binary alloys, so that the order of the α phase to α_2_ could start at higher temperatures, even before γ was formed. This could result in smaller lamella distances, which stabilized the lamellar structure of the α_2_+γ colonies. Due to the preferred order of α_2_ compared to γ, the resulting α_2_ phase fraction was increased by higher oxygen contents [37,38,39,40]. Due to large differences in oxygen solubility between the α_2_ and γ phase, the conversion from α to γ was significantly slowed down.

During heat treatment at 1290 °C, the γ phase was solved when T_γ-solvus_ was reached. When cooling from the (α + β) phase field, the known solid-state transformations took place. Again, γ lamellae were formed from supersaturated α grains [41]. During further cooling, due to the phase imbalance, supersaturated α2 grains segregated to lamellar α2/γ colonies. At the same time, a reaction according to α2 → β0 + γ took place, also to compensate for the phase imbalance. The γ lamellae coarsened until they formed globular γ grains. The main driving force for the forming process is the minimization of interfacial energy [42]. At low cooling rates, α_2_ grains were not supersaturated, and the formation of the fine-lamellar structures did not occur. In Figure 1a, as described in [30], parts of coarse γ lamellae are visible. From this, it can be concluded that for DED samples, the γ phase had not been completely dissolved, and, therefore, the local T_γ-solvus_ temperature was >1290 °C. The increase in T_γ-solvus_ could be attributed to (locally) higher Al contents or increased oxygen contents. Residues of α_2_ grains were coherent with the comparatively slow cooling during furnace cooling. The boundaries of the lamellar α2+γ grains in the heat-treated cast sample, depicted in Figure 1d, were also occupied by γ and β_0_ phase. The heat treatment temperature of 1290 °C was thus also for the elemental composition of the cast material, despite the low Al content, locally below T_γ-solvus_. The same was valid for the microstructure of the forged sample in Figure 1c. The morphology of the γ grains was slightly different from the cast state; due to the forging process, some grains were elongated. In comparison, the microstructure of the LPBF samples (Figure 1b) was almost completely lamellar, and some grain boundary areas with globular γ phase and very small portions β_0_ were present. The heat treatment temperature (1290 °C) was, therefore, ≥T_γ-solvus_ for the large, completely lamellar microstructure components of the LPBF material.

Overall, Figure 1 implies that except for the LPBF sample, all samples were heat treated in the (α + γ) phase field, and T_γ-solvus_ was not exceeded at least proportionally. The composition alone didn’t deliver a valid argument for this exception. From this, it can be deduced that the transformation processes during the heat treatment were also significantly influenced by the very fine LPBF microstructure in the as-built condition. The original grain size is about 1–7 µm diameter and, thus, approximately 1/3 the size of DED and 10 times smaller compared to cast [28]. Many interfaces and short diffusion paths thus represented an essential influencing variable.

#### 3.2.2. Phase Composition

In Table 4, measurement results from the synchrotron HEXRD investigation are listed. All materials investigated showed at least three phases, with significant differences in the respective phase distribution, despite the chemical similarity of the materials.

The γ phase fraction in DED samples was higher than in LPBF, cast, and forged samples. The β_0_ phase content in both LPBF and DED samples was smaller than in cast or forged samples. This went along with the observations from Figure 1 and, together with Table 3, suggested a correlation between γ phase and aluminum content, on the one hand, and fineness of the structure and β_0_ phase fraction, on the other. However, local fluctuations in the chemical composition and smaller differences in the fineness of the microstructure, which are also present in the other material samples, must be considered to a limited extent. The formation of γ grains is generally favored because of the discontinuous coarsening in fine-grained structures, which is described in, e.g., [43].

**Figure 2:**
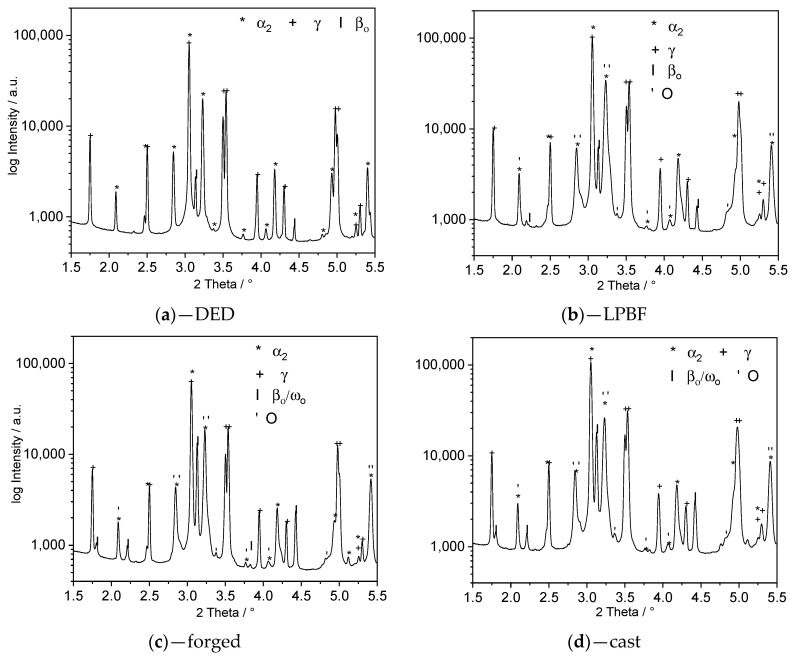
Diffraction patterns of heat-treated TNM (**a**) manufactured by DED; (**b**) manufactured by LPBF; (**c**) manufactured by forging; (**d**) manufactured by casting.

In addition to the aforementioned, the existence of further phases (O and ω_0_) can be read from the diffractograms in Figure 2. Both phase fractions were very small, and a clear separation for quantification was not possible because of overlapping peaks of the crystallographic-related phases.

The ω_0_ phase was formed as nanoscale precipitations from β/β_0_ at temperatures around 900 °C. During heating up, the ω_0_ phase dissolved in the temperature range of 930–960 °C, and the β_0_ phase was present [33]. The orthorhombic O phase was visibly formed from α_2_-phase at temperatures below 660 °C, remained stable up to that temperature, and was associated with improved room temperature ductility [44,45]. The O phase was dissolved again at about 700 °C. The occurrence of both phases could be explained by cooling during heat treatment. By slow furnace cooling with approx. 4 K/s, the material reached the respective temperature range of the formation of the ω_0_ and O phase for a sufficiently long time. Since the α_2_-phase fraction in DED material was relatively small compared to the other samples, the associated O phase formation didn’t take place to a detectable amount. The same could be derived for the ω_0_ phase, whose occurrence was observed in the samples with a comparatively high β-content produced by casting and forging.

### 3.3. Mechanical Properties

#### High-Temperature Compressive Strength

In Table 5, the results of the compression tests, performed at 700 °C on three samples each, are listed. 

It can be seen that, on average and taking into account the deviation, both compressive strength, measured on the basis of the fracture strength, and ductility of the DED specimens were comparable with the characteristic values of the cast and forged specimens. The range of elastic deformation ended consistently at approx. 500–550 MPa S_d0,2_. The fracture strength S_B_ was approx. 1150–1200 MPa. The fracture compression A_t_ of all materials except for the DED specimens was approx. 15.3%. The comparatively greater fracture compression of the material built up with DED could be attributed to the fine-grained nature of the material, as it can be seen from Figure 1. This, again, was highly dependent on the parameters used for the sample production [32]. 

### 3.4. Repair and Hybrid Manufacturing

#### 3.4.1. Feasibility

**Figure 3:**
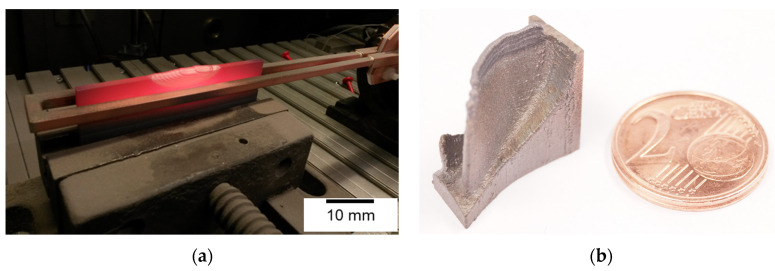
Photographs of hybrid γ-TiAl samples. (**a**) DED and cast, (**b**) DED and LPBF.

In Figure 3, photographs of hybrid samples are presented. These showed the feasibility of repair and hybrid manufacturing of γ-TiAl.

In Figure 3a, a mockup cast part with a DED repaired section is depicted in as-built condition, cooling down immediately after the process. In Figure 3b, a section of an LPBF turbocharger wheel with a DED tip is presented to demonstrate the possible combination of these processes as well.

#### 3.4.2. Microstructure of Hybrid Materials

**Figure 4:**
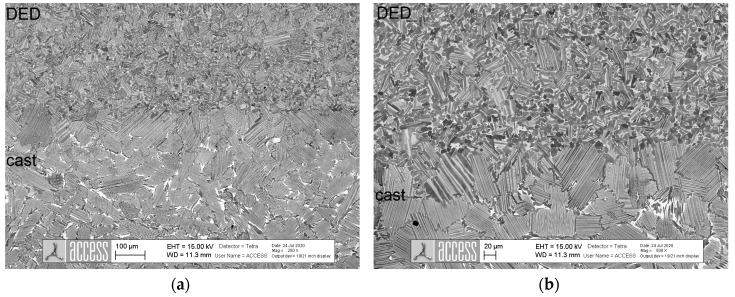
SEM images of the transition zone between cast and DED material in heat-treated condition (**a**) 100×; (**b**) 500× magnification.

For further investigation, hybrid samples consisting of a cast base and a DED upper section of a total size of 60 mm (length) × 20 mm (height) × 4 mm (width) were produced, heat-treated, and analyzed. In Figure 4, SEM images of the transition zone between the cast (bottom) and DED (top) material in heat-treated conditions are depicted.

The materials could be clearly distinguished by their grain sizes and phases. The lamellar grains at the direct transition (heat affected zone) also appeared larger than those of the pure cast material. The heat influence of the application of the first DED layers was, therefore, large enough to activate grain growth already during the build-up process. Stresses were well absorbed by the material; no crack formation occurred.

#### 3.4.3. Micro Indentation of Hybrid Materials

In Table 6, the results of the micro indentation tests, performed on four hybrid samples with three indents each, are listed. Despite the generally high scattering, higher hardness values were measured in the cast material.

**Figure 5:**
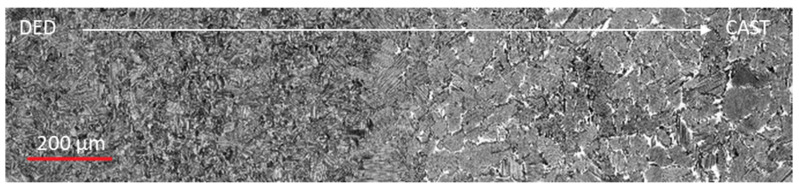
Micro indentation test zone of hybrid γ-TiAl samples. DED (**left**) and cast (**right**).

In Figure 5, an exemplary SEM image of the test zone of one hybrid sample is added. The transition area itself was rather small at approx. 200 µm and could be identified by the differences between the grain sizes.

An explanation was provided by the proportion of (fine) lamellar areas in the test surface, which was significantly larger in the cast material. DED material had superfine lamellae as well as very small grain sizes. Both spoke for high hardness values. As the test indentations were relatively large, the probability of hitting purely lamellar grains was higher in the cast material, so that, on average, the softer phases were measured in the DED area. In the hybrid zone, the proportion of the β phase was again somewhat smaller than in the cast area, so that the hardness was still slightly higher, but just as high as in the cast material when deviations were taken into account.

## 4. Discussion

The high-temperature compressive strength (700 °C) of DED-manufactured material is approx. 1200 MPa, which is also comparable to that of the identically heat-treated cast, forged, and LPBF material. The biggest differences are found in the almost non-existent β_0_ phase and an approx. 15% higher γ phase content compared to cast, forged, and LPBF material, which can be attributed to an approx. 0.4% higher aluminum content of the DED samples. Since the oxygen content of all tested materials is similarly high at approx. 2000 ppm, this can be ruled out as the main cause of the differences between the materials. The overall low strength values for TNM-B1 can be explained both by the possibly less favorable heat treatment and the high oxygen content. Thus, the properties of γ-TiAl produced with DED correspond, in the context of the investigations carried out, overall to those achieved with processes used to produce components made of γ-TiAl.

The oxygen content of all samples is above the limit value of 1200 ppm. This allows for a more specific analysis of other influences in this study but is known to be too high for application purposes [46]. The ductility, especially at room temperature, is highly influenced by O, which is detrimental for at least any post-processing. For cast and thus forged material, O contents are regularly lower, usually well below 800 ppm [47]. Samples with higher content are deliberately selected in order to make the composition comparable and to be able to observe the influence of the different temperature-time curves of the production routes. For AM (Additive Manufacturing), the processing steps of powder production and the additive manufacturing itself will always lead to an unavoidable increase in oxygen. However, EBM (Electron Beam Melting) already allows us to minimize the uptake during AM close to zero due to the vacuum, which is why it is the most frequently selected AM method for processing γ-TiAl. At the same time, aluminum loss is comparatively higher [48]. The resulting EBM microstructures [49], are quite like the ones achieved with LPBF in this work, which suggests a transferability of the results shown.

The hardness of hybrid DED and cast samples varies measurably depending on the test area.

Contrary to other measurements, the hardness of the cast area is higher than that of the DED area in this test [30,32]. Due to the small grain sizes of the three-phase DED material, the positioning and size of the measuring points are particularly important. The transition zone shows the high lamellar microstructure of the cast with partly smaller grain sizes of DED so that there is a tendency to slightly higher hardness.

Due to the similarity of the material properties and the flawless transition zones, DED is generally suitable to be combined with AM, casting, or forging. The ability to build on 3D structures allows for repair and modification or individualization of series production parts likewise. 

## 5. Conclusions

The investigated DED samples differ uniformly from material samples produced by other methods in that their aluminum content is at least 0.4% higher, which can easily be balanced when considered in the powder production process. As a consequence, the DED T_γ-solvus_ temperature is increased. Therefore, the selected heat treatment (1290°, 1 h, FC) is carried out at too low a temperature to completely equalize the microstructure of TNM-B1 materials of different production routes. This is likely to be improved with an increase in the heat treatment temperature. However, despite the differences in microstructure, the mechanical properties are quite similar to a fracture strength of approx. 1200 MPa.

The process-related feasibility of combining DED with other manufacturing methods in a hybrid process is demonstrated. The results overall imply that DED can likely be used as a repair and hybrid manufacturing technique for γ-TiAl parts. Interesting in the future and a consistent next step towards industrial implementation are investigations of mechanical properties of such compounds, the targeted adjustment of which can represent a further step towards the more efficient exploitation of material properties.

## Figures and Tables

**Table 1:** The process parameters for DED (Direct Energy Deposition) of TNM^TM^ (Ti-Nb-Mo) [32].

Beam Diameter	Laser Power	Scanning Velocity	Offset (z)
0.6 mm	66 W	500 mm/min	0.25 mm

**Table 2:** The process parameters for LPBF (Laser Powder Bed Fusion) of TNM^TM.^

Beam Diameter	Laser Power	Scanning Velocity	Layer Thickness
0.09 mm	200 W	1200 mm/s	0.03 mm

**Table 3:** Chemical composition of TNM-B1 samples; values in wt.-%.

Sample	Ti	Al	Nb	Mo	B	O ^1^
DED	58.59	29.14	9.6	2.15	0.038	2130
LPBF	60.38	28.67	8.26	2.06	0.036	1770
Cast	60.41	28.19	8.85	2.24	0.021	1750
Forging	60.32	28.35	8.73	2.19	0.025	2070

^1^ values given in ppm.

**Table 4:** The phase composition of heat-treated TNM-B1 samples; values in vol.-%.

Sample	α_2_/(O)	γ	β_0_/(ω_0_)
DED	22.9	75.8	1.3
LPBF	40.6	57.4	2.0
Cast	29.5	63.9	6.6
Forging	34.7	57.7	7.6

**Table 5:** Compressive strength of TNM-B1 samples at 700 °C.

Sample	Compressive Yield Strength S_d0.2_	Fracture Strength S_B_	Compression at Break A_t_
DED	566 MPa ± 23	1190 MPa ± 44	24.0 %
LPBF	465 MPa ± 49	1237 MPa ± 10	15.1 %
Cast	551 MPa ± 22	1219 MPa ± 15	15.4 %
Forging	567 MPa ± 15	1210 MPa ± 15	15.4 %

**Table 6:** Microhardness HV-30 of TNM-B1 samples.

Zone	Mean Value	Standard Deviation
DED	347	22
Hybrid	387	28
Cast	383	27
